# Parkin regulates drug-taking behavior in rat model of methamphetamine use disorder

**DOI:** 10.1038/s41398-021-01387-7

**Published:** 2021-05-17

**Authors:** Akhil Sharma, Arman Harutyunyan, Bernard L. Schneider, Anna Moszczynska

**Affiliations:** 1 Department of Pharmaceutical Sciences, Eugene Applebaum College of Pharmacy and Health Sciences, Wayne State University, 259 Mack Ave, Detroit, MI 48201 USA; 2 College of Pharmacy, Natural and Health Sciences, Manchester University, 10627 Diebold Rd, Fort Wayne, IN 46845 USA; 3 Brain Mind Institute, École Polytechnique Fédérale de Lausanne School of Life Sciences, Ch. Des Mines, 9, CH-1202 Geneve, Switzerland

**Keywords:** Addiction, Genomics

## Abstract

There is no FDA-approved medication for methamphetamine (METH) use disorder. New therapeutic approaches are needed, especially for people who use METH heavily and are at high risk for overdose. This study used genetically engineered rats to evaluate PARKIN as a potential target for METH use disorder. PARKIN knockout, PARKIN-overexpressing, and wild-type young adult male Long Evans rats were trained to self-administer high doses of METH using an extended-access METH self-administration paradigm. Reinforcing/rewarding properties of METH were assessed by quantifying drug-taking behavior and time spent in a METH-paired environment. PARKIN knockout rats self-administered more METH and spent more time in the METH-paired environment than wild-type rats. Wild-type rats overexpressing PARKIN self-administered less METH and spent less time in the METH-paired environment. PARKIN knockout rats overexpressing PARKIN self-administered less METH during the first half of drug self-administration days than PARKIN-deficient rats. The results indicate that rats with PARKIN excess or PARKIN deficit are useful models for studying neural substrates underlying “resilience” or vulnerability to METH use disorder and identify PARKIN as a novel potential drug target to treat heavy use of METH.

## Introduction

Methamphetamine (METH) use disorder is a worldwide health problem. In the United States, there are close to 2,000,000 people who use METH, and deaths from METH overdose are rapidly rising^1,2^. Despite numerous clinical trials conducted to date, there is no FDA-approved medication for METH use disorder. The medications tested in clinical trials have shown low efficacy in people who use METH moderately and no effect in those who use METH heavily^3–5^. New therapeutic approaches are needed, particularly for people who use METH heavily, as they suffer the most from METH abuse-related neuropsychological problems^6–8^, are less likely to seek treatment than those using the drug moderately^9^, and are at high risk of dying from METH overdose^10^. Early intervention in METH abuse by lowering METH intake is essential not only for preventing METH overdose but also for subsequent interventions, as greater treatment participation is achieved when METH use is low^9^.

METH use disorder has been linked to alterations in dopaminergic and glutamatergic neurotransmission, alterations in energy metabolism and cytoskeletal arrangement as well as to oxidative stress and inflammation^11–24^. Protein-ubiquitin ligase PARKIN may be a potential novel drug target in METH use disorder as its function has been linked to these processes^25–31^. Despite evidence for a potential role of PARKIN in METH use disorder, ours is the first study linking PARKIN to this disorder. We used genetically engineered rats to evaluate PARKIN as a potential target for METH use disorder, namely PARKIN-deficient (PKO), PARKIN-overexpressing (PO), and wild-type (WT) young adult male Long Evans rats. PARKIN was overexpressed in the nucleus accumbens because the nucleus accumbens is involved in multiple phases of the development of compulsive self-administration of stimulants, including its initiation, development, and maintenance^32^ and because it is the hub between emotion, motivation, and action^33^.

Drug addiction is a chronic, relapsing disorder that has been characterized by compulsive seeking and escalated intake of drugs. In the current study, were trained rats to self-administer high doses of METH using an extended-access METH self-administration (EA METH SA) paradigm with three ratio schedules of reinforcement. This paradigm produced an escalation of METH intake with rats working much harder to obtain METH with an increasing ratio schedule and, therefore, modeled important aspects of human drug abuse (compulsive drug taking, cravings, and dependence)^34^ resulting in heavy consumption of METH seen in some people dependent on METH. Furthermore, similar to people who abuse METH, rats with extended access to intravenous METH demonstrate cognitive deficits^35^. Rats were allowed to self-administer high METH doses for 10 days. Reinforcing properties of METH were assessed by measuring the number of lever presses for METH and METH intake during each of ten operant sessions. Rewarding properties of METH were assessed by measuring time spent in a METH-paired environment. We demonstrate that PKO rats are predisposed to heavy METH use compared to WT counterparts whereas rats overexpressing PARKIN in the nucleus accumbens are less predisposed.

## Materials and methods

### Animals

**Fig. 1:**
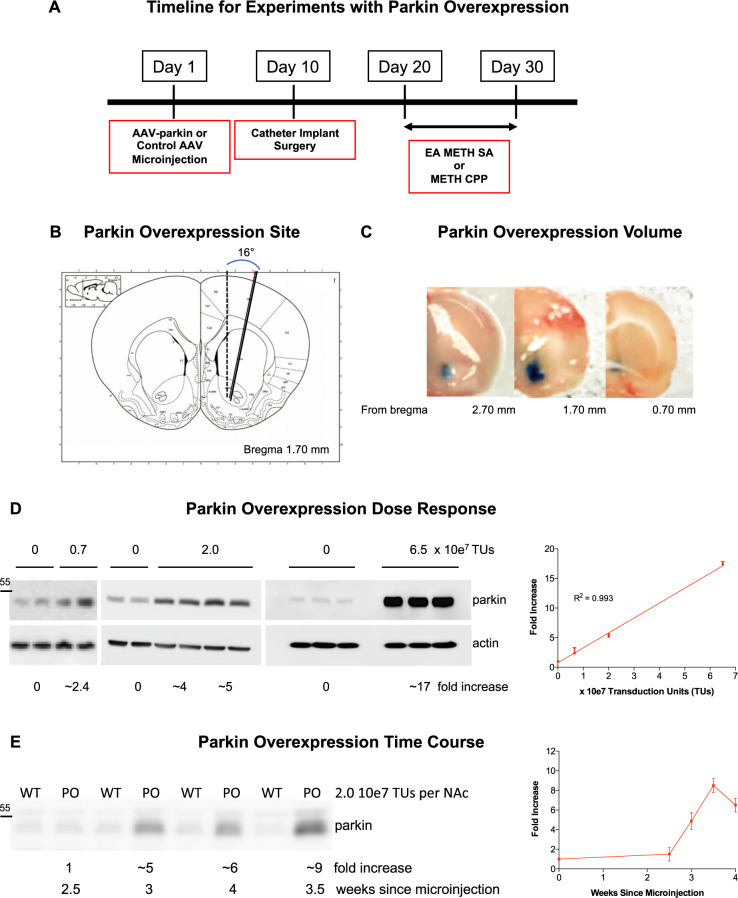
Validation of PARKIN overexpression. **A** Timeline for experiments with PARKIN overexpression. **B**, **C** Validation of the overexpression site (nucleus accumbens, 0.7–2.7 mm from bregma) and spread (medial-lateral, dorsal-ventral, and anterior-posterior). **D** Dose–response of PARKIN overexpression (0, 0.7, 2, or 6.5 × 10^7^ TUs/side) in the nucleus accumbens (*n* = 3 or 4/group). The data followed a linear regression curve (*R*^2^ = 0.993, *p* < 0.0001). **E** Time course of PARKIN overexpression (2 × 10^7^ TUs/side) showing that PARKIN overexpression reached the maximum at around 3.5 weeks after the gene transfer vector microinjection and excess of PARKIN persisted at least for the duration of the study (~4 weeks) (**D**). Parkin band migrated to ~52 kDa (relate to the marked molecular weight of 55 kDa). Parkin band was the only band detected; a representative full blot is presented in Fig. 4.

The study employed young adult male Long Evans rats (~55 days old at the beginning of the study, *N* = 177) of four genotypes: WT rats (Harlan, Indianapolis, IN, USA), PKO rats (*Park2*^−/−^, SAGE Labs, Missouri, MO, USA), PO rats, and PKO PO rats. PO rats were generated by bilaterally overexpressing PARKIN in the nucleus accumbens of WT rats whereas PKO PO rats were generated by bilaterally overexpressing PARKIN in the nucleus accumbens of PKO rats. The stereotaxic surgery was performed as previously published^36^, with some modifications. Microinjections were done at a 16° angle into these coordinates: +1.8 A/P, ±3.2 M/L, −7.6 D/V from bregma. Validation of PARKIN protein loss in PKO rats is shown in supplementary Fig. S1, whereas validation of PARKIN overexpression is shown in Fig. 1. PARKIN was not present in PKO samples as assessed by sodium dodecyl sulfate-polyacrylamide gel electrophoresis/western blotting. Upon arrival, animals were pair-housed and maintained under standard environmental conditions in an AAALAC-accredited vivarium. Animals were maintained on a 12 h light/dark cycle with ad libitum access to food and water unless specified otherwise. Bodyweight was monitored on a daily basis before and during experiments (Suppl. Fig. S2). No method of randomization into groups was used. All experiments were approved by the Wayne State University Institutional Animal Care and Use Committee and conducted in compliance with the ARRIVE guidelines.

### Extended-access methamphetamine self-administration (EA METH SA) and conditioned place preference test

**Fig. 2:**
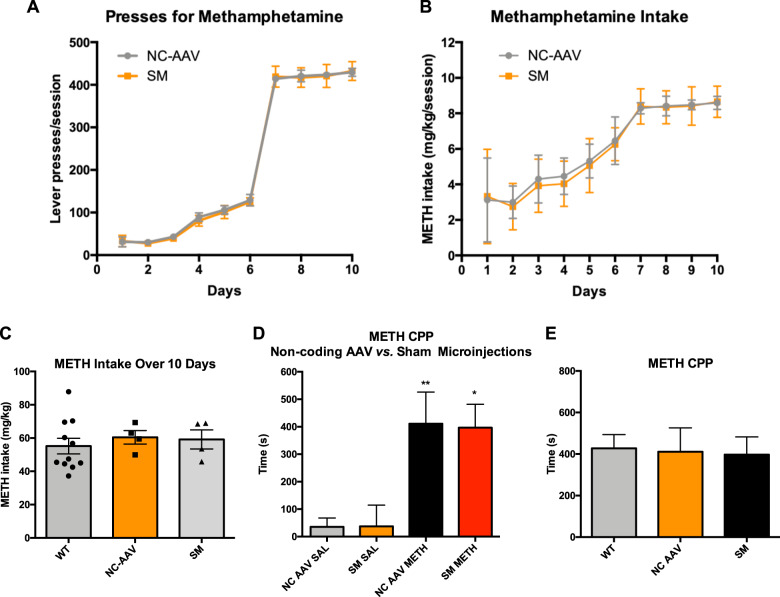
Noncoding-AAV and Sham-microinjection control for behavioral experiments. Neither noncoding AAV2/6 (NC-AAV) nor sham microinjection (SM, microinjection of phosphate-buffered saline) had an effect on methamphetamine (METH) self-administration or preference for METH-paired compartment in the conditions place preference test in wild type (WT) rats. **A** Lever presses in NC-AAV and SM group. **B** METH intake in NC-AAV and SM group. **C** Conditioned place preference test results for NC-AAV and SM group. **D** METH intake in WT vs. NC-AAV and SM rats. **E** Preference for METH-paired compartment in WT vs. NC-AAV and SM rats. Data are expressed as mean ± SEM, *n* = 4/group. Abbreviations: CPP conditioned place preference, NAc nucleus accumbens, L left, R right.

In the first experiment, PKO and WT rats were trained to self-administer high doses of METH (measurement of METH reinforcement) (Fig. 1A) or subjected to the conditioned place preference test (measurement of METH reward) (Fig. 2A). In the second experiment, the reinforcing/rewarding properties of METH were assessed in PO rats as compared to WT rats. In the third experiment, PARKIN was overexpressed in the nucleus accumbens of PKO rats that were subsequently compared to non-overexpressing PKO rats (phenotype rescue experiment). During EA METH SA, rats had access to METH (0.1 mg/kg/injection) for 15 h/day for 10 days at increasing fixed ratio (FR) schedule: FR1 (days 1–3), FR2 (days 4–6), and FR5 (days 7–10). During METH conditioning for conditioned place preference test, rats were injected with 4 mg/kg METH or saline on alternate days.

Supplementary Online Data provides extensive details on stereotaxic surgery as well as the EA METH SA and conditioned place preference.

### Electrophoresis and western blotting

Rats were sacrificed 10 days after the last operant session. Nucleus accumbens was punched out of 2 mm-thick coronal brain section encompassing 0.7–2.7 mm from bregma. Electrophoresis and western blotting on nucleus accumbens pieces were performed as previously described^37^, utilizing anti-PARKIN and anti-β-ACTIN (1:1000, 2132S, and 3700S, respectively, Cell Signaling Technology, Danvers, MA, USA) primary antibodies. Immunoreactivities were quantified using ImageJ software (National Institutes of Health, Bethesda, MD, USA) and presented as relative optical densities normalized to control samples.

### Statistical analyses

The experimental data sets were analyzed using IBM SPSS v.25 (IBM, Armonk, NY). EA METH SA data were analyzed by two-way mixed-design ANOVA whereas METH conditioned place preference data were analyzed by two-way factorial ANOVA, both with Bonferroni correction. Before each ANOVA, the data were examined for the presence of outliers using an IQR of 3. After the removal of outlier(s) the data were examined for normality (Shapiro–Wilk’s test), equality of variances (Levene’s test), and sphericity (Mauchly’s test). When Mauchly’s test was statistically significant, a Greenhouse–Geisser correction was employed. Group differences in PARKIN levels or total METH intake were assessed with the unpaired two-tailed Student’s *t* test. Group sizes were estimated based on published behavioral data. To assess sizes of genotype-mediated effects and probabilities of the presence of the effects, partial eta square (*η*_p_^2^) and power of analysis (1*-β*) values were calculated. Statistical significance was set at *p* ≥ 0.05. Pearson’s correlation analysis was performed on PARKIN overexpression data. Body weight data were analyzed by repeated measure ANOVA with Bonferroni post hoc test. All data, with the exception of body weight data, are reported as mean ± SEM. Body weight data are reported as mean ± SD (Supplementary data, Fig. S2). The investigators were not blinded to the group allocation during the experiments or when assessing the outcome.

## Results

### Validation of PARKIN overexpression

Viral vector microinjections took place 3 weeks before METH self-administration or conditioned place preference experiments to allow for maximal PARKIN overexpression (Fig. 1A). Investigation of the microinjection sites using Trypan blue dye confirmed that during microinjections, the needle reached approximately the middle of the nucleus accumbens (Fig. 1B) and that 2 μL of the dye did not spread beyond the borders of the nucleus accumbens (Fig. 1B, C). Administration of 0.7 × 10^7^, 2 × 10^7^, or 6.5 × 10^7^ TUs/side of AAV2/6-*parkin* consistently produced a 1.8–3, 4–6, or 15–20-fold increase, respectively, in PARKIN levels in WT rats; the data followed linear regression curve (*R*^2^ = 0.993, *p* < 0.0001) (Fig. 1D). PARKIN overexpression reached the maximum at around 3.5 weeks after the gene transfer vector microinjection and lasted till the end of the behavioral experiments (~4 weeks) (Fig. 1E).

We next assessed whether noncoding AAV2/6 or sham microinjection (SM) of saline has an effect on the outcome of EA METH SA or METH conditioned place preference test. Rats were bilaterally microinjected with noncoding AAV2/6 or 2 μL sterile phosphate-buffered saline into the nucleus accumbens. These groups were named noncoding-AAV (NC-AAV) group and SM group, respectively. Both groups underwent EA METH SA and METH conditioned place preference test. During METH self-administration, both NC-AAV and SM rats pressed for METH at a similar rate (main effect of microinjection type (noncoding AAV2/6 or saline): *F*_(1,6)_ = 0.015, *p* = 0.908, *η*_p_^2^ = 0.002, 1*-β* = 0.051; main effect of time: *F*_(9,54)_ = 101, *p* < 0.001, *η*_p_^2^ = 0.994, 1*β* = 1.0; *n* = 4/group) (Fig. 2A). Concordant with this result, neither microinjection of noncoding AAV2/6 nor microinjection of saline significantly influenced METH intake (main effect of microinjection type: *F*_(1,6)_ = 0.033, *p* = 0.862, *η*_p_^2^ = 0.005, 1-*β* = 0.053; main effect of time: *F*_(9,54)_ = 62.2, *p* < 0.001, *η*_p_^2^ = 0.912, 1*-β* = 1.0; *n* = 4/group) (Fig. 2B). Total consumption of METH (over 10 days of EA METH SA) by WT, NC-AAV, and SM group is presented in Fig. 2C.

During the conditioned place preference test, both NC-AAV and SM rats displayed a preference for the METH-paired compartment (main effect of treatment: *F*_(1,12)_ = 19.6, *p* < 0.001, *η*_p_^2^ = 0.620, 1-*β* = 0.982, *n* = 4/group; NC-AAV: *F*_(1,12)_ = 10.2, *p* < 0.01, *η*_p_^2^ = 0.460, 1-*β* = 0.835; SM: *F*_(1,12)_ = 9.36, *p* < 0.05, *η*_p_^2^ = 0.438. 1-*β* = 0.802) and did not differ in respect to time spent in this compartment (*p* > 0.1) (Fig. 2D). Similarly, these experimental groups did not significantly differ in respect to time spent in the saline-paired compartment (*p* > 0.1). There was no statistically significant interaction between treatment and microinjection type on METH-paired place preference (*p* > 0.1). Comparison of conditioned place preference data for WT, NC-AAV, and SM groups is presented in Fig. 2E. In summary, the NC-AAV and SM groups did not differ from non-microinjected WT rats in respect to self-administered METH or degree of preference for the METH-paired compartment. This data indicated that neither noncoding AAV2/6 nor SM influenced the outcomes of EA METH SA or METH conditioned place preference test.

### PARKIN knockout increases extended-access METH self-administration

**Fig. 3:**
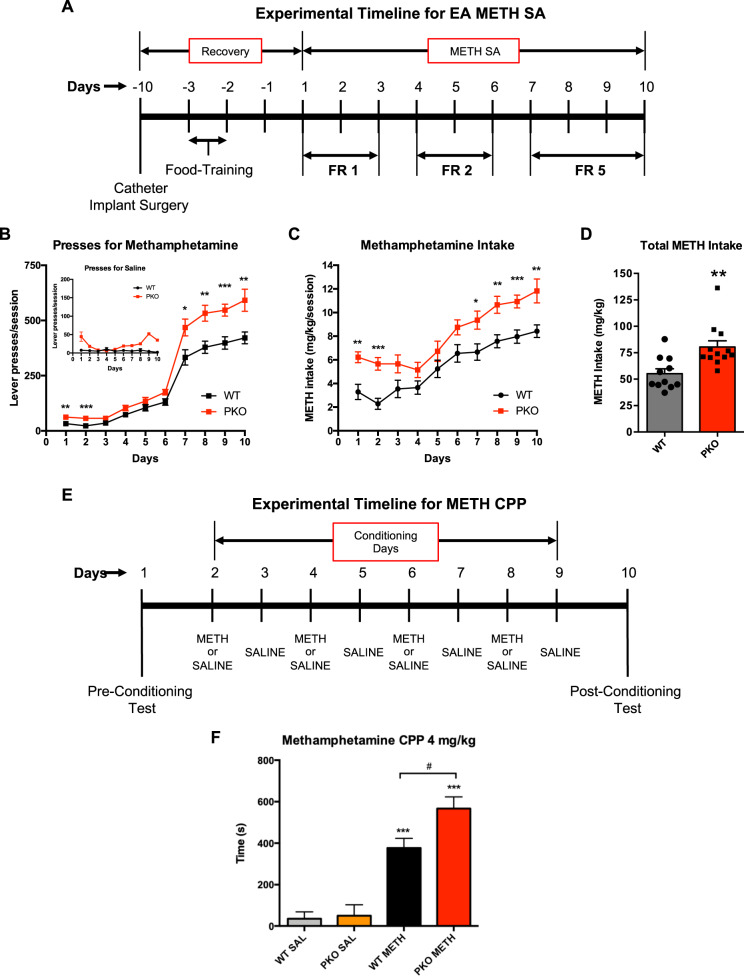
PARKIN knockout increases extended-access methamphetamine self-administration (EA METH SA) and METH reward. **A** Timeline of EA METH SA experiment. **B**, **C** Escalation of METH taking in *Parkin* gene knockout (PKO) rats as compared to wild-type (WT) rats during an increasing ratio schedule of reinforcement: FR1, FR2, and FR5. The PKO rats pressed for METH at a significantly higher rate than the WT rats (**B**) with a consequent higher consumption of METH (**C**). There was no significant difference between PKO and WT rats in respect of the number of presses for saline (**B** inset, *n* = 4/group). *p < 0.05, **p < 0.01, ***p < 0.001, *n* = 11/group. **D** Total amount of METH consumed by PKO rats was significantly higher than that consumed by WT rats (+39% **p < 0.001). **E** Timeline of conditioned place preference experiment. **F** The PKO rats displayed a significantly higher preference for METH (4 mg/kg) than the WT rats. ***p < 0.001 as compared to the respective saline (SAL) controls, ^#^*p* < 0.05 WT vs. PKO, *n* = 6/group. Data are expressed as mean ± SEM. Abbreviations: *CPP* conditioned place preference, *FR* fixed ratio.

To determine whether PKO rats are more dependent on METH as compared to WT rats, the rats from both genotypes were subjected to the EA METH SA test (Fig. 3A). Both WT and PKO rats showed an escalation of METH self-administration (Fig. 3B, C) The PKO rats weighed more than WT rats but lost weight at the same rate as WT rats during EA METH SA (Fig. S2A). Including body weight as a covariate in ANOVA analysis showed that it did not influence METH self-administration (*F*_(1,19)_=0.100, *p* = 0.756, *η*_p_^2^ = 0.005, 1-*β* = 0.06, *n* = 11/genotype). There was a significant main effect of genotype (*F*_(1,20)_ = 16.7, *p* < 0.01, *η*_p_^2^ = 0.454, 1-*β* = 0.973) as well as time (*F*_(2.9,58)_ = 322, *p* < 0.001, *η*_p_^2^ = 0.942, 1-*β* = 1.0) on lever presses for METH, and significant interaction between the two factors (*F*_(2.9,58)_ = 5.40, *p* < 0.01, *η*_p_^2^ = 0.213, 1-*β* = 0.913). Pairwise comparisons revealed that PKOs pressed more than WTs during the first two sessions (*p* < 0.01 and *p* < 0.001, respectively) and during the compulsive phase of METH self-administration (day 7, *p* < 0.05 and days 8–10, *p* < 0.01) (Fig. 3B). Concordant with this result, genotype and time both significantly influenced METH intake (genotype: *F*_(1,20)_ = 13.8. *p* < 0.01, *η*_p_^2^ = 0.409, 1-*β* = 0.941; time: *F*_(3.6,71)_=53.8, *p* < 0.001, *η*_p_^2^ = 0.729, 1-*β* = 1.0), but there was no significant interaction between the two factors (*F*_(3.6,71)_ = 1.40, *p* = 0.246). The pairwise comparisons revealed that PKO rats self-administered significantly more METH during the first two sessions (*p* < 0.01 and *p* < 0.001, respectively) and during the compulsive phase of METH self-administration (day 7, *p* < 0.05 and days 8–10, *p* < 0.01) (Fig. 3C). There was no significant difference between PKO and WT rats in respect to the number of presses for saline (Fig. 3B inset, *n* = 4/genotype), confirming that METH, not the light stimulus, acted as a reinforcer. The total amount of METH consumed by PKO rats was significantly higher than that consumed by WT rats (+39%, *p* < 0.001) (Fig. 3D).

The results from the EA METH SA indicate that METH exerts stronger reinforcing effects in rats lacking PARKIN than in WT controls. The effect of PARKIN knockout on METH self-administration was of large size (large *η*_p_^2^) and significant, thus strongly suggesting PARKIN as a suppressor of reinforcing effects of METH.

### PARKIN knockout increases preference for METH-paired compartment

Compulsive drug use is maintained by the rewarding properties of the drug and by the loss of control over drug intake. To determine whether PARKIN knockout increases the rewarding effects of METH, PKO, and WT rats underwent the conditioned place preference test (Fig. 3E). Though the conditioned place preference test has limitations^38^, it provides information about the rewarding effect of contextual cues associated with a drug stimulus. Two-way ANOVA revealed a trend for significant interaction between genotype and treatment (*F*_(1,20)_ = 3.31, *p* = 0.084, *η*_p_^2^ = 0.142, 1-*β* = 0.410), and main effects of both genotype and treatment on METH preference, with the effect of treatment being stronger than the effect of genotype (*F*_(1,20)_ = 79.2, *p* < 0.001, *η*_p_^2^ = 0.798, 1-*β* = 1.00, and *F*_(1,20)_ = 4.50, *p* < 0.05, *η*_p_^2^ = 0.184, 1-*β* = 0.523, respectively, *n* = 6/group). Pairwise comparisons revealed that there was no significant difference in baseline preference for a compartment between PKO and WT rats (*p* = 0.834) (Fig. 3F). Both genotypes showed a significant METH-induced conditioned place preference (*p* < 0.001), with the PKO rats displaying a significantly higher preference for the METH-paired compartment than the WT rats (*p* < 0.05) (Fig. 3F). ANCOVA determined that bodyweight had no influence on the results (not shown).

The results from the conditioned place preference test suggest that METH-paired contextual cues are more rewarding for PKO rats than for WT rats.

### Extended-access METH self-administration leads to PARKIN deficit in the nucleus accumbens

**Fig. 4:**
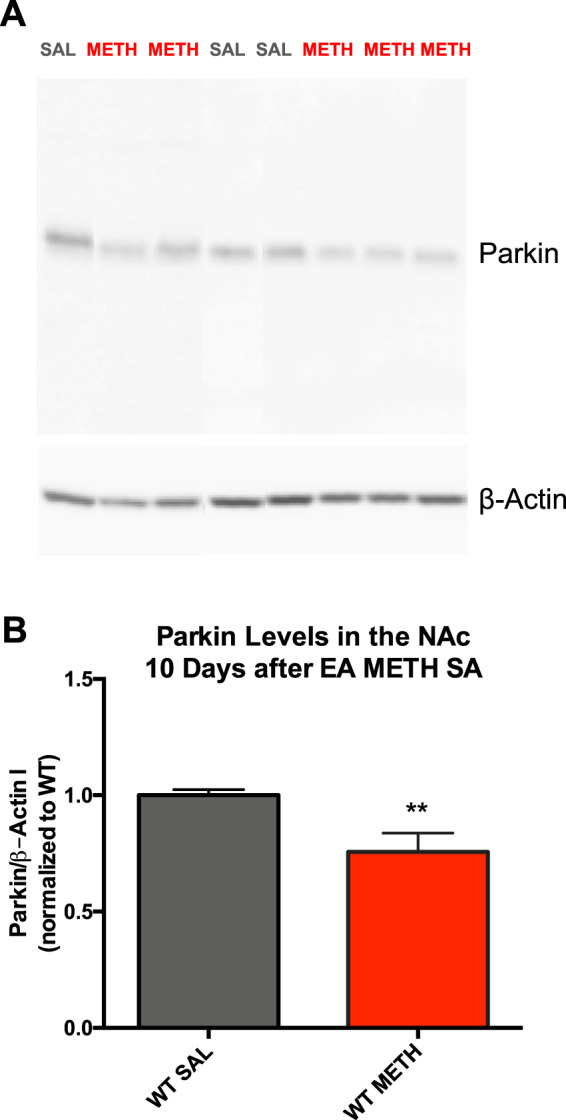
Extended-access methamphetamine self-administration (EA METH SA) decreases PARKIN protein levels in the nucleus accumbens. **A** Representative PARKIN bands (~52 kDa) assessed in nucleus accumbens (NAc) of wild type (WT) rats, with β-ACTIN serving as a loading control: SAL (black) represents saline-yoked WT rats while METH (red) represents WT rats that underwent EA METH SA; both group of rats were killed 10 days after the last operant session. **B** Quantification of PARKIN bands. As compared to saline (SAL) administration, EA METH SA led to a deficit (−24%) in PARKIN levels in the nucleus accumbens of WT rats at 10 days after the last operant session. **p < 0.01, *n* = 8 or 9/group. Data are expressed as mean ± SEM.

We previously determined that METH binge decreases PARKIN levels in the striatum via oxidative damage^39^. To determine whether exposure of WT rats to the EA METH SA paradigm decreases PARKIN levels in the nucleus accumbens, PARKIN levels were assessed in rats that self-administered METH at 10 days after the last operant session and compared to PARKIN levels in rats exposed to saline. METH-exposed rats had significantly lower levels of PARKIN in the nucleus accumbens as compared to saline-exposed rats (−24%, *t* = 3.02, *df* = 15, *p* < 0.01, *n* = 8 or 9) (Fig. 4A, B).

### WT rats overexpressing PARKIN self-administer less METH and spend less time in the METH-paired compartment than WT controls

**Fig. 5:**
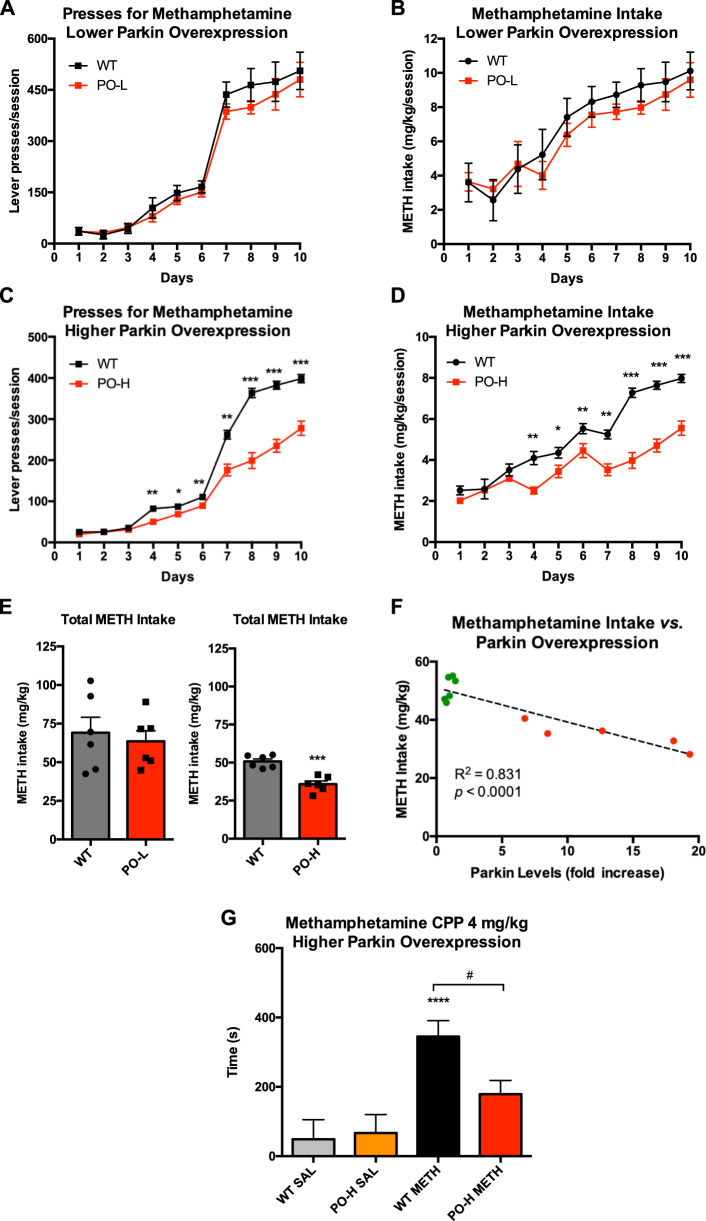
PARKIN overexpression in the nucleus accumbens of WT rats attenuates extended-access methamphetamine self-administration (EA METH SA) and METH reward. Escalation of METH taking in rats overexpressing PARKIN in the nucleus accumbens as compared to non-expressing wild-type (WT) rats during an increasing ratio schedule of reinforcement: FR1, FR2, and FR5. **A** At the low PARKIN overexpression (4–6-fold), the PARKIN overexpressing (PO) rats did not press for METH at a significantly lower rate than the WT rats and, consequently, **B** they did not consume less METH than the WT rats. **C** At the high PARKIN overexpression (15–20-fold), the PO rats did press for METH at a significantly lower rate than the WT rats and **D** consumed less METH than the WT rats. *p < 0.05, **p < 0.01, ***p < 0.001, WT vs. PO-H, *n* = 6/group. **E** Quantification of total METH intake in PO-L and PO-H rats as compared to their respective WT control groups. There was no significant difference in total METH intake between the PO-L group and WT controls whereas the PO-H group consumed significantly less METH than the WT group (−33%, ***p < 0.001). **F** Correlation analysis of total METH intake with PARKIN levels in PO-H group (red dots: PO-H group; green dots: WT group). **G** The 15–20-fold excess of PARKIN was sufficient to significantly attenuate preference for METH (4 mg/kg) in WT rats in the conditioned place preference test. ***p < 0.001 as compared to the respective saline controls, ^#^*p* < 0.05 WT vs. PO-H, *n* = 6/group. Data are expressed as mean ± SEM. Abbreviations: *NAc* nucleus accumbens, *FR* fixed ratio, *PO-L* lower PAKIN overexpression, *PO-H* higher PARKIN overexpression, *SAL* saline.

Since WT rats subjected to EA METH SA had lower PARKIN levels in the nucleus accumbens than saline controls, we next investigated whether overexpression of PARKIN in the nucleus accumbens of WT rats would result in attenuated METH self-administration as compared to non-overexpressing WT rats. Two concentrations of AAV2/6-*parkin* were utilized to overexpress PARKIN—2 × 10^7^ or 6.5 × 10^7^ TUs/side (usulally producing 4–6 or 15–20-fold excess of PARKIN). The PO rats weighed slightly less than WT controls (Fig. S2B); however, body weight did not have a significant effect on METH self-administration as a covariate (*F*_(1,9)_ = 0.006, *p* = 0.939, *η*_p_^2^ = 0.001, 1-*β* = 0.051, *n* = 6/group). At low levels of PARKIN overexpression, there was a significant main effect of time (*F*_(3.1,31.5)_ = 51.6, *p* < 0.001, *η*_p_^2^ = 0.838, 1-*β* = 1.0), but not of genotype (*p* > 0.1), on lever presses for METH (Fig. 5A). There was no statistically significant interaction between the two factors (*p* > 0.1). Statistical analysis of METH intake over 10 days produced a similar result: there was a significant effect of time (*F*_(1.54,15.4)_ = 187, *p* < 0.001, *η*_p_^2^ = 0.949, 1-*β* = 1.0), but no significant main effect of genotype (*p* > 0.1) and no significant genotype × time interaction (*p* > 0.1) (Fig. 5B). Ten days after the last operant session, the average PARKIN overexpression in PO-L group was 3.8-fold (not shown). There was no statistically significant correlation between total METH intake and PARKIN levels (*R*^2^ = 0.077, *p* = 0.382).

At high levels of PARKIN overexpression, there was a strong significant effect of genotype *F*_(1,10)_ = 47.8, *p* < 0.0001, *η*_p_^2^ = 0.827, 1-*β* = 1.0), as well as time (*F*_(2.19,21.9)_ = 543, *p* < 0.0001, *η*_p_^2^ = 0.982, 1-*β* = 1.0), on METH lever pressing (Fig. 5C) and significant interaction between these variables (*F*_(2.19,21.9)_ = 34.9, *p* < 0.0001, *η*_p_^2^ = 0.577). Pairwise comparisons revealed that PO rats pressed significantly less for METH on days 4–10, with the strongest effect at the compulsive stage of METH abuse (day 4, *p* < 0.001; days 5 and 6, *p* < 0.05; day 7 *p* < 0.001; days 8–10, *p* < 0.0001) (Fig. 5C). In concordance, there was a strong significant effect of genotype (*F*_(1,10)_ = 31.6, *p* < 0.0001, *η*_p_^2^ = 0.760, 1-*β* = 0.999) as well as time (*F*_(4,40)_ = 103, *p* < 0.0001, *η*_p_^2^ = 0.912, 1-*β* = 1.0) on METH intake and significant interaction between these variables (*F*_(4,40)_ = 14.4, *p* < 0.0001, *η*_p_^2^ = 0.573). As with lever presses, pairwise comparisons showed that POs took significantly less METH on days 4–10, with the strongest effect on days 8–10 (day 4, *p* < 0.001; days 5 and 6, *p* < 0.05; day 7, *p* < 0.001; days 8–10, *p* < 0.0001) (Fig. 5D). Figure 5E presents quantification of total METH intake in PO-L and PO-H rats as compared to their respective WT control groups. There was no significant difference in total METH intake between the PO-L group and WT controls, whereas the PO-H group consumed significantly less METH than the corresponding WT controls (−33%, ***p < 0.001). Ten days after the last operant session, the average PARKIN overexpression in the PO-H group was 16-fold (6–19 fold) (Fig. 5F). Pearson’s correlation analysis revealed that total METH intake by the PO-H group highly and significantly correlated with PARKIN levels in the nucleus accumbens (*R*^2^ = 0.831, *p* < 0.0001) (Fig. 5F).

WT rats overexpressing PARKIN at high levels spent less time in the METH-paired compartment in the conditioned preference test than non-overexpressing WT controls (Fig. 5G). Two-way ANOVA revealed the main effect of treatment on METH preference (*F*_(1,20)_ = 17.1, *p* < 0.001, *η*_p_^2^ = 0.461, 1-*β* = 0.976) and a trend for significant interaction between genotype and treatment (*F*_(1,20)_ = 3.47, *p* = 0.077, *η*_p_^2^ = 0.148, 1-*β* = 0.426). Pairwise comparisons detected no significant difference in baseline preference for a compartment between PO and WT rats (*p* = 0.800, *n* = 6/group). Significant preference for the METH-paired compartment, as compared to the saline-paired compartment, was detected in WT rats (*p* < 0.0001) but not in PO rats (*p* = 0.123), however, there was a difference between genotypes in the METH group (*p* < 0.05) (Fig. 5G). The ANCOVA analysis determined that bodyweight had no influence on the results (not shown).

These results further support the conclusion that PARKIN can negatively regulate reinforcing/rewarding properties of METH if its levels are increased at least sixfold.

### PARKIN knockout rats overexpressing PARKIN self-administer less METH during the first half of drug self-administration compared to PARKIN-deficient rats

**Fig. 6:**
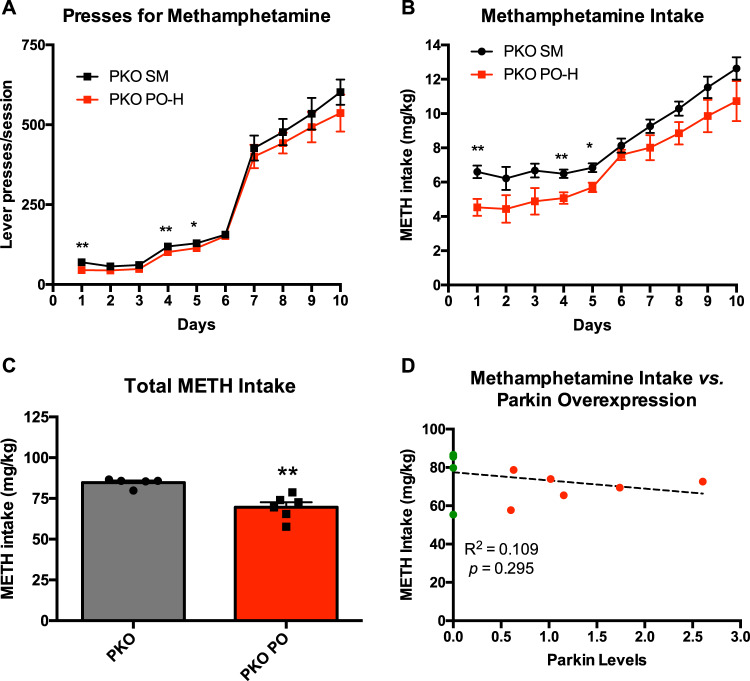
The effect of PARKIN overexpression in the nucleus accumbens of *Parkin* gene knockout (PKO) rats. High PARKIN overexpression (15–20-fold) in the nucleus accumbens of *Parkin* knockout PKO rats significantly attenuated methamphetamine (METH) taking in the first half of the extended-access METH self-administration as compared to non-overexpressing PKO rats. **A** Lever presses for METH, **B** METH intake. **C** Total amount of METH consumed by PKO PO rats was significantly lower than the total amount of METH consumed by PKO rats (−18%). **D** Correlation analysis of total METH intake with PARKIN levels in PKO PO group (red dots: PKO PO group; green dots: PKO group). *p < 0.05, **p < 0.01, *n* = 5 or 6/group. Data are expressed as mean ± SEM.

The next experiment aimed to determine whether overexpression of PARKIN in the nucleus accumbens of PKO rats would attenuate METH self-administration as it did in the nucleus accumbens of WT rats. High levels (15–20-fold) of PARKIN were overexpressed in the nucleus accumbens of PKO rats (*n* = 5 or 6/group), while control rats were microinjected with saline (SM surgery). PKO and PKO PO rats did not differ in respect to body weight during the 10 days of METH SA (Fig. S2C). There was a significant main effect of time (*F*_(1.2,11)_ = 192, *p* < 0.0001, *η*_p_^2^ = 0.838, 1-*β* = 1.0) and a weaker main effect of genotype (*F*_(1,9)_ = 5.15, *p* = 0.05, *η*_p_^2^ = 0.364, 1-*β* = 0.525) on lever presses for METH between the genotypes (Fig. 6A). There was no statistically significant interaction between the time and genotype (*p* > 0.1). Examination of METH intake data produced similar results: a significant main effect of time (*F*_(1.5,13.4)_ = 29.3, *p* < 0.0001 0.765, 1-*β* = 1.0) and genotype (*F*_(1,9)_ = 18.3, *p* < 0.01, *η*_p_^2^ = 0.671, 1-*β* = 0.966) but no significant genotype × time interaction (*p* > 0.1). Pairwise comparisons revealed that PKO PO rats pressed significantly less for METH and, consequently, ingested significantly less of the drug than non-overexpressing PKO rats in the first half of EA METH SA (days 1, 4, and 5) (*p* < 0.01, *p* < 0.01, and *p* < 0.001, respectively) but not at later stages of EA METH SA (Fig. 6B). The total amount of METH consumed by PKO PO rats was significantly lower than the total amount of METH consumed by PKO rats (−18%, *p* < 0.01) (Fig. 6C). There was no statistically significant correlation between total METH intake by the PKO PO group and PARKIN levels in the nucleus accumbens measured at 10 days after the last operant session (*R*^2^ = 0.109, *p* = 0.295) (Fig. 6D).

This result suggests that PARKIN knockout-induced compensatory changes may have lessened the effect of PARKIN overexpression.

## Discussion

METH use disorder is a disorder without pharmacotherapy and, therefore, in need of new drug targets for the development of new drugs. In this study, we used genetically engineered rats to evaluate PARKIN as a potential drug target for METH use disorder. We found that PARKIN knockout rats self-administered more METH and spent more time in the METH-paired environment than WT rats whereas WT rats overexpressing PARKIN in the nucleus accumbens at least sixfold self-administered less METH and spent less time in the METH-paired environment. We also found that PARKIN knockout rats overexpressing PARKIN in the nucleus accumbens self-administered less METH during the first half of drug self-administration days than PARKIN-deficient rats. The results indicate that rats with PARKIN excess or PARKIN deficit are useful models for studying neural substrates underlying “resilience” or vulnerability to METH use disorder and identify PARKIN as a novel potential drug target to treat heavy use of METH.

### The role of PARKIN in METH-taking behavior

Drug-taking behavior is a consequence of reinforcing effects of the drug of abuse, mediated by goal-directed and habit systems in the brain. At the initial stages of drug self-administration, the goal-directed system directs the relationship between action (lever pressing) and outcome (drug infusion and experiencing sought-after drug effects). After prolonged drug-taking, instrumental action becomes compulsive and habitual^40^. Escalation of drug self-administration is a hallmark of compulsive drug taking and drug dependence^34^. Hence, escalation of METH self-administration by both PKO and WT rats despite the increasing effort required to obtain METH (from 1 to 5 presses needed to obtain METH injection) suggests increasing motivation to obtain the drug, leading to METH dependence. Compared to the WT controls, the PKO rats pressed significantly more for METH and, consequently, consumed more METH than WT rats during the initial stage (FR1) and during the FR5 stage (compulsive intake) of EA METH SA. In contrast, the PO rats pressed significantly less for METH and consumed more of the drug at these stages. Furthermore, METH intake highly correlated with the levels of PARKIN in the nucleus accumbens of WT rats overexpressing PARKIN 6–19-fold. Two conclusions can be reached based on these findings. First is that PARKIN may be involved in negative modulation of reinforcing/rewarding effects of METH within the nucleus accumbens, which is the main brain region mediating rewarding/reinforcing effects^41^. This conclusion is supported by the results from the conditioned place preference test. The conditioned place preference test and self-administration measure different processes, namely the rewarding effects of a drug through context-drug associative learning *vs*. reinforcing effects of a drug through operant conditioning, respectively. However, there is reasonable concordance between drugs that produce conditioned place preference and drugs that are self-administered^38^. Because both paradigms involve associative learning (albeit of a different type)^38,42^ and because the nucleus accumbens plays a role in this types of learning^40,43–45^, PARKIN may have a role in processes associating METH effects with the context or operant behavior. In addition to playing a key role in mediating reward, the nucleus accumbens controls goal-directed behavior during instrumental conditioning^40,46–49^. Consequently, the second conclusion is that accumbal PARKIN may negatively regulate goal-directed behavior during METH self-administration. We demonstrated that the volume spread after the microinjections were largely confined to the nucleus accumbens. Therefore, it is unlikely that the difference in METH self-administration observed between PO and WT rats was due to unspecific PARKIN overexpression in the adjacent dorsomedial striatum, which is a key brain area regulating goal-directed behavior^40,50–52^, but rather to overexpression of PARKIN in the nucleus accumbens, which has a role in action selection^49^. Habitual drug-taking is mediated by the dorsolateral striatum^40,50,53,54^ and it is not known whether striatal PARKIN affects this behavior. Overexpression of PARKIN in the nucleus accumbens likely resulted in its excess not only in accumbal perikarya but also in terminals located in brain areas to which the nucleus accumbens projects to^36^. Consequently, PARKIN could have presynaptically modulated METH taking in these brain areas. Even though PKO and PO rats displayed opposite addictive behaviors, molecular mechanisms underlying these behaviors may be different due to compensatory mechanisms that occurred in the brains of PKO but not in PO rats during development^55^. Such changes might explain the smaller effect of PARKIN overexpression on METH taking in PKO PO rats as compared to PO rats. Alternatively, total loss of PARKIN was harder to overcome than the moderate PARKIN deficit (−24%) induced by METH SA in WT rats.

### Molecular determinants of altered METH-taking behavior in PKO and PO rats

PARKIN is a ubiquitous ubiquitin-protein ligase with neuroprotective and anti-inflammatory properties^56–58^, mostly known for its link to Parkinson’s disease^59^. The canonical function of PARKIN is to polyubiquitinate proteins destined for degradation by proteasome or lysosome (short-lived, misfolded, or damaged proteins)^26,60^. PARKIN also has degradation-independent functions, which it exerts by monoubiquitination of its substrates^60^. To date, about three dozen PARKIN substrates have been identified in various cellular compartments, including mitochondrial, endoplasmic reticulum, and synaptic proteins, that belong to various signaling pathways^61,62^.

In relation to the present investigation, studies in young adult PKO mice (3–4-month-old) reported no change in the levels of striatal dopamine or dopamine receptors as compared to WT littermates^63,64^, with dopamine metabolites reported unchanged or elevated^65,66^. Our previous study in young adult PKO rats (2-month-old) similarly found no change in dopamine or dopamine metabolites. PKO rats had normal levels of dopamine transporter; however, PARKIN may modulate dopaminergic neurotransmission *via* ubiquitination of the transporter and regulation of its trafficking^67,68^. Dopamine D2 receptor has not been identified as a substrate for PARKIN; however, we found that PKO rats had lower levels of the glycosylated form of postsynaptic D2 receptor than WT rats in the dorsal striatum^37^. Low D2/D3 receptor availability in striatal regions, including the nucleus accumbens, is considered a risk factor for METH use disorder because it is a molecular feature of impulsivity; animal, as well as human data, support this notion^69,70^. Furthermore, it has been demonstrated that D2-expressing medium spiny neurons in the nucleus accumbens antagonize the reinforcing and rewarding effects of another psychostimulant, cocaine^71^. *Parkin* gene knockout results in augmented glutamatergic neurotransmission and altered levels of glutamate receptors as well as γ-aminobutyric acid B (GABA_B)_ receptors in mice^31^. This data suggests the involvement of PARKIN protein in the regulation of these receptors at the synapse. In conclusion, PARKIN deficit-mediated alterations in dopaminergic, glutamatergic, and/or GABAergic neurotransmission likely modulate the rewarding/reinforcing effects of METH via their receptors^72–77^.

METH use disorder has been linked to alterations in energy metabolism and cytoskeletal arrangement as well as to oxidative stress and inflammation^11–21,78–80^. For example, METH-induced inflammation in the nucleus accumbens was shown to mediate METH reinforcing properties^11,78^. In the EA METH SA paradigm, large doses of METH were self-administered, likely causing oxidative stress and inflammation in multiple brain areas, including the nucleus accumbens^11,81^. PARKIN function has been linked to energy metabolism, and cytoskeletal arrangement as well as to protection against oxidative stress, and inflammation^25,26,29–31^. Therefore, PARKIN is well-positioned to regulate responses to METH and could have decreased METH intake in rats via any of the aforementioned mechanisms.

Addiction circuitry includes multiple brain areas^82^, all of which have likely undergone neuroadaptive changes in the PKO rats during development^55^. We hypothesize that because of these changes and lack of PARKIN in addiction circuitry, overexpression of PARKIN in the nucleus accumbens only had a smaller effect on METH self-administration in PKO rats than WT rats. Overexpression of PARKIN in the nucleus accumbens and prefrontal cortex and/or dorsal striatum may be necessary to suppress the addictive phenotype in PKO rats. Data from PKO PO rats suggest that PARKIN has a stronger effect on molecular mechanisms underlying reinforcing properties than on those underlying loss of control over METH intake in these rats. It remains to be determined whether PARKIN overexpression in the nucleus accumbens of PKO rats would have a more pronounced effect on METH self-administration in a paradigm with short access to the drug (e.g., 1 h), consequently leading to low METH intake.

## Limitations and methodological considerations

A limitation of using PKO rats is that developmental adaptations to the absence of the *Parkin* gene may have compensated for an early loss of gene function. As a result, such adaptations may have obscured the analysis of the effects of PARKIN protein per se. Another limitation of our study is different environments in which the PKO and WT rats spent the first 2 months of their lives (different vendors), a factor that can affect gene expression and, consequently, phenotype. Nevertheless, overexpression of PARKIN resulted in behaviors opposite to those observed in PKO rats, thus validating the role of PARKIN in METH self-administration. A high level of PARKIN overexpression was needed for the desired effect, which can be viewed as non-translational. However, since we employed a model of very high METH intake, lower levels of PARKIN overexpression will likely work with models of moderate METH intake.

PARKIN activity is regulated by Zn^2+^, an array of posttranslational modifications as well as by interactions with a variety of regulatory proteins^83,84^. Several research groups have been working on increasing PARKIN activity in relation to Parkinson’s or Alzheimer’s disease. These studies range from manipulation of *Parkin* gene to manipulation of upstream and downstream PARKIN interactors. Some studies focus on relieving autoinhibitory interactions within PARKIN. Our laboratory investigates the possibility of increasing PARKIN levels by manipulation of proteins downstream of PARKIN substrates. Currently, there is no effective PARKIN activator for in vivo use despite these research efforts. The efforts continue and, therefore, increasing PARKIN expression or activity pharmacologically may be possible in near future.

## Conclusions

To date, pharmacological approaches have failed as treatments for METH use disorder. We demonstrated for the first time that PARKIN plays important role in this disorder. Specifically, we demonstrated that PARKIN modulates the rewarding/reinforcing properties of high-dose METH. The role of PARKIN in drug use disorder has not been previously studied; therefore, our study is novel. From the public health point of view, the most important finding of this investigation is that overexpression of PARKIN in the nucleus accumbens alone was sufficient to attenuate intake of very high doses of METH by WT rats, making PARKIN a novel potential drug target to treat heavy use of METH. In addition, we demonstrated that rats with PARKIN excess or PARKIN deficit are useful models for studying neural substrates underlying “resilience” or vulnerability to METH use disorder.

## Supplementary information

Supplementary Data
